# The genetic variation of mitochondrial sequences and pathological differences of *Echinococcus multilocularis* strains from different continents

**DOI:** 10.1128/spectrum.01318-24

**Published:** 2025-02-14

**Authors:** Baoping Guo, Gang Guo, Wenjing Qi, Malike Aizezi, Chuanchuan Wu, Mengxiao Tian, Adriano Casulli, Wenbao Zhang, Jun Li

**Affiliations:** 1 State Key Laboratory of Pathogenesis, Prevention and Treatment of High Incidence Diseases in Central Asia, Clinical Medicine Institute, The First Affiliated Hospital of Xinjiang Medical University, Urumqi, Xinjiang, China; 2 Xinjiang Clinical Research Center for Perinatal Diseases, Urumqi Maternal and Child Health Hospital, Urumqi, Xinjiang, China; 3 Xinjiang Uyghur Autonomous Region Center for Animal Disease Control and Prevention, Urumqi, Xinjiang, China, Urumqi, China; 4 Basic Medical College, Xinjiang Medical University, Urumqi, Xinjiang, China; 5 WHO Collaborating Centre for the Epidemiology, Detection and Control of Cystic and Alveolar Echinococcosis, Istituto Superiore Di Sanità, Rome, Italy; Hubei University of Medicine, Shiyan, China

**Keywords:** alveolar echinococcosis, *Echinococcus multilocularis*, mitochondrial, pathological lesions, strains, phylogenetic analysis, evolutionary distance

## Abstract

Alveolar echinococcosis is a lethal zoonotic disease caused by the fox tapeworm *Echinococcus multilocularis*. The parasite is widely distributed in the Northern Hemisphere and exhibits low genetic diversity among populations. To compare the differences among four *E. multilocularis* strains from different geographical locations, namely, Alaska (EM-AK), Japan (EM-JP), Xinjiang (EM-XJ), and Ningxia (EM-NX), their complete mitochondrial (mt) sequences were compared, and their induced pathological lesions were analyzed in mouse models. The complete mt sequence of EM-AK resulted in 0.84%–0.86% variation as compared with the other strains, which had a lower variation. Phylogenetic analysis and parsimony network indicated that EM-AK resulted in 30,000 years of evolutionary distance from the other three strains. EM-AK induced more pathological damage than the other three strains, which was likely to induce more host cell infiltration and acute granuloma in the liver. More importantly, EM-AK produced more protoscoleces than the other three strains, which may impact the transmission dynamics of the parasite. Given the geographical location of four strains, which is far from each other, and also the pathological differences, the strains of *E. multilocularis* are likely models for addressing the relationship and interfacial immune response between the host and the helminth.

**IMPORTANCE:**

*Echinococcus multilocularis* is the causative agent of alveolar echinococcosis, which is considered the most serious parasitic disease in the Northern Hemisphere. There are many genotypes, but the pathogenic and mitochondria sequence and differences are still unclear. Therefore, this study showed both pathological and genetic differences between the four strains of *E. multilocularis*. EM-AK induced more severe immune responses and especially induced more host cell infiltration, which resulted in more severe granuloma in the liver. EM-JP has metacestode lesions morphologically closer to those of *E. granulosus* with clear cyst fluid. However, this strain produced much fewer protoscoleces (PSCs). Genetically, EM-AK is more distant from other strains.

## INTRODUCTION

Alveolar echinococcosis (AE) caused by the fox tapeworm *Echinococcus multilocularis* is a potentially lethal zoonotic parasitic disease (1, 2) since its proliferative progression is similar to a slow-growing liver cancer (3, 4). AE is found in the Northern Hemisphere (5, 6), including Northern America, Europe, and Central Asia. The fatality rate of AE reaches up to 90% without proper treatment (7). AE is highly endemic in the western part of China, including Sichuan, Xinjiang, Ningxia, Qinghai, Gansu, Tibet, and Inner Mongolia (8–10). Among the estimated 18,235 new AE cases per year globally, 91% occur in China (9), where the prevalence of human AE can be even greater than 3–5% in some areas (11).

*E. multilocularis* exhibits genetic differences based on the variation of its mitochondrial (mt) DNA (in particular when considering *cox1* and *nad1* genes), which are likely associated with different geographic locations of specimens collected from central Europe, Alaska, and Kazakhstan (12–15). However, the classification may be insufficient to indicate the real evolutionary relationships among these geographical clades of *E. multilocularis*, as it relied only on the three mt gene fragments, namely, *cox1*, *nad1*, and *atp6*. Recent studies using the complete mt sequences determined a reliable evolutionary relationship among genotypes (16, 17).

Although mt sequences of *E. multilocularis* have been reported (13, 18, 19), only one complete mt genomes (OR911426) has been published in China (17). In addition, there are no reports on the sequence variation of the whole mt genomes. In this article, we compared the whole mt genome sequences of the following four strains: *E. multilocularis* Alaska (EM-AK), Japan (EM-JP), Xinjiang (EM-XJ), and Ningxia (EM-NX) originally collected from the aforementioned endemic areas. We also showed the different pathological features of originating from these from these different geographical areas.

## RESULTS

### Intraperitoneal pathology of four *Echinococcus multilocularis* strains in Kunming (KM) mice

**Fig 1:**
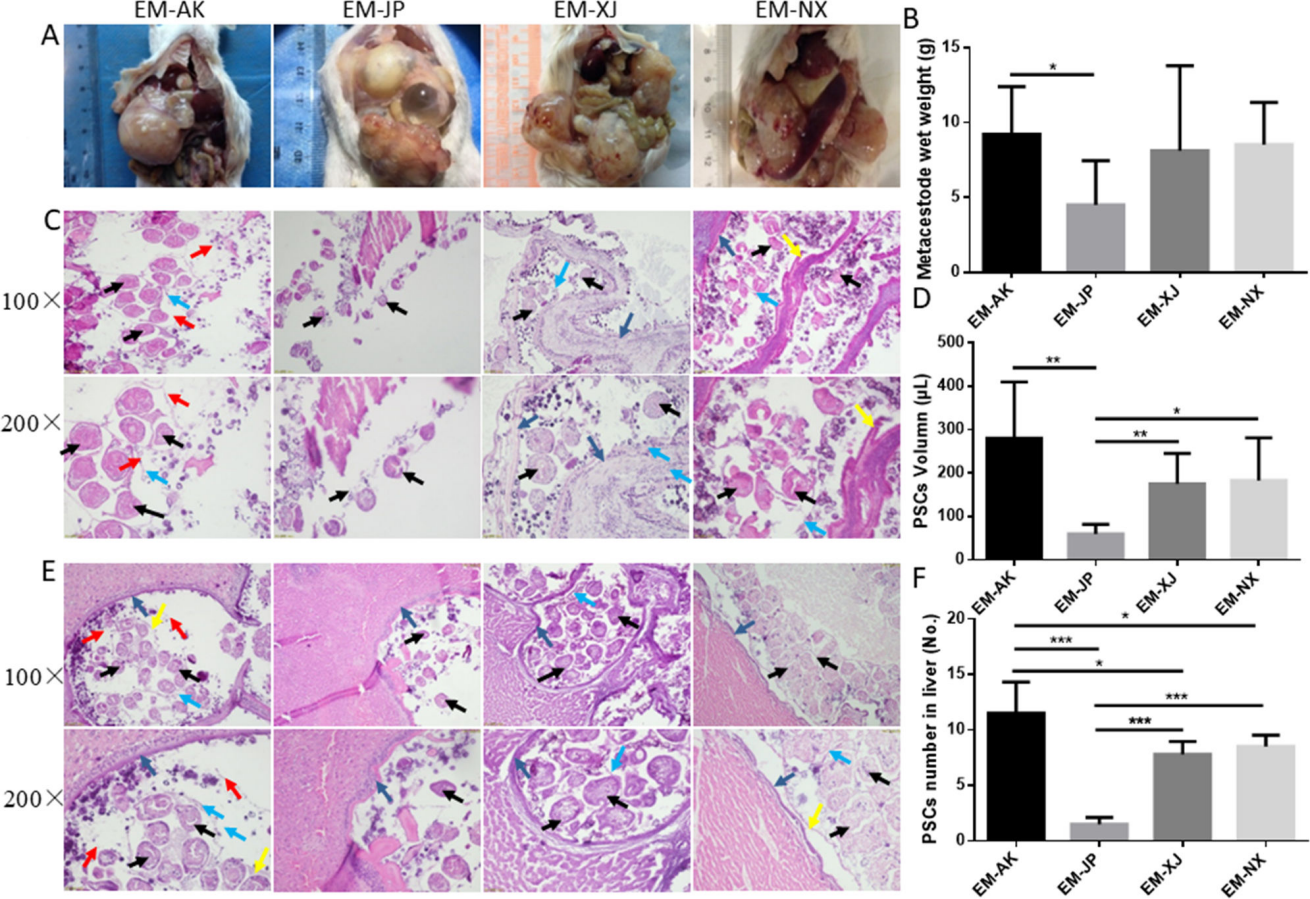
Metacestodes induced pathology by intraperitoneal injection of protoscoleces (PSCs) from four different *Echinococcus multilocularis* strains in Kunming (KM) mice (*n* = 7–9 mice per group). (**A**) Morphology of metacestodes 4 months after infection. (**B**) Metacestode wet weight. (**C**) H&E staining images of metacestodes (×100 and ×200). (**D**) Volume of precipitated PSCs. (**E**) H&E staining images showing metacestodes penetrating the liver through intraperitoneal cavity (×100 and ×200). (**F**) Average PSC numbers in each H&E section field of the liver. Note: the black arrow indicates PSCs, and the red arrow indicates the capsule wall. EM-AK, Alaska strain; EM-JP, Japan strain; EM-XJ, Xinjiang strain; EM-NX, Ningxia strain; H&E, hematoxylin and eosin; and PSCs, protoscoleces.

Although the mice were inoculated by intraperitoneal injection of PSCs, the metacestodes were found not only in the abdominal cavity but also in the surface of liver, chest cavity, submesenteric adipose tissues, and other organs, with some vesicles penetrating into the paranchyma of these organs (Table S1). The successful infection rate (number of mice developing metacestodes/total mice inoculated with PSCs of *E. multilocularis*) was 87.50, 55.60, 75, and 71.42% for the EM-AK, EM-JP, EM-XJ, and EM-NX strains, respectively. However, the *χ* test analysis indicated no significant difference (*P* > 0.05) (Table S1). The metacestodes of EM-AK were granular, yellowish, and opaque, but those of EM-JP had a cyst-like form with thin and translucent cysts containing clear cyst fluid (5). In contrast, the metacestodes of the other three strains had thick outer layers with much less parasitic fluid (Fig. 1A). Hematoxylin and eosin (H&E) staining revealed that EM-AK induced higher metacestode weight and a greater number of PSCs in the metacestode than other strains (*P* < 0.05) (Fig. 1B) with more severe pathological responses in the lesions.

After 4 months of growth in the peritoneal cavity of the mice, differences in the metacestodes wet weight, PSC production, and pathological lesion were recorded among the four strains. Figure 1 shows the volume and number of PSCs and lesions in the mice. EM-AK produced more PSCs and had a higher metacestode weight than the other three strains. Strain EM-JP produced significantly fewer PSCs (Fig. 1C). However, strain EM-JP produced cyst-like metacestodes or micro-vesicles, which are like *E. granulosus* cysts with clear cyst fluid (20). Strain EM-AK (9.22 ± 2.89) higher number of PSCs per lesion area than strain EM-JP (4.51 ± 2.82) (*P* < 0.05). However, compared with EM-XJ (8.12 ± 5.07) and EM-NX (8.53 ± 2.57), the difference was not significant (vs. EM-XJ, *P* > 0.05; vs. EM-NX, *P* > 0.05).

The precipitation volume of PSCs (μL) of the EM-AK (280.31 ± 116.62) strain in mice was higher than that of the EM-JP (60 ± 20.00), EM-XJ (175 ± 66.14), and EM-NX (183.33 ± 89.75) strains. EM-JP produced a significantly lower volume of PSCs indicating reduced production of PSCs (Fig. 1B and C). A large number of PSCs was also observed in infected liver samples, and the results (number of PSCs per area) indicated that EM-AK (11.52 ± 2.51) produced more PSCs than EM-JP (1.50 ± 0.54), EM-XJ (7.80 ± 1.04), and EM-NX (8.5 ± 0.96), with a significant difference (vs. EM-JP, *P* < 0.001; vs. EM-XJ, *P* < 0.05; vs. EM-NX, *P* < 0.05). EM-AK had brood capsules, each containing three to 10 PSCs, whereas the EM-XJ and EM-NX strains had fewer brood capsules in KM mice (Fig. 1D). There was no brood capsule found in EM-JP lesions.

### Liver pathological difference among the four strains of *E. multilocularis*

**Fig 2:**
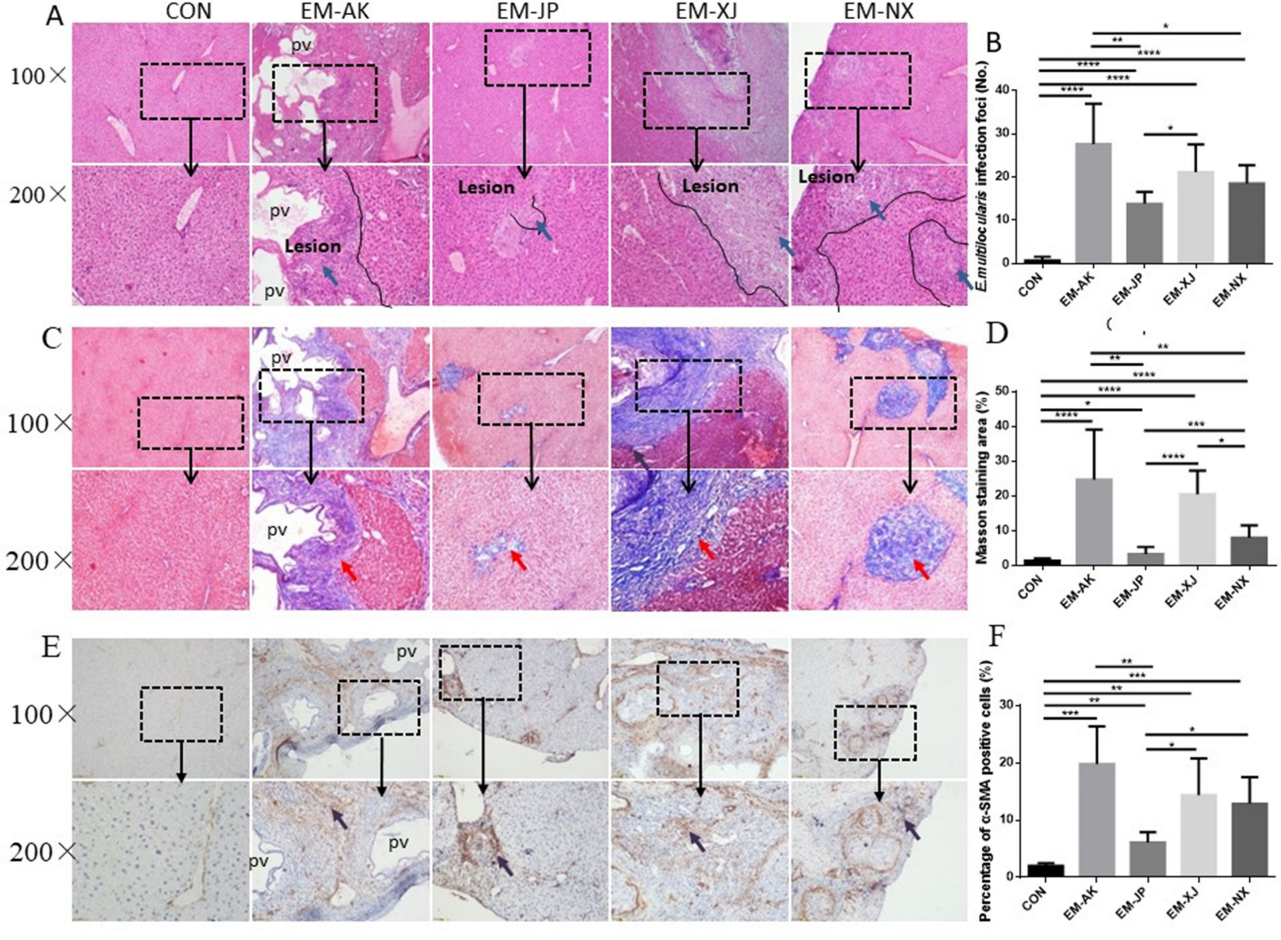
Hepatic histopathological analysis of C57BL/6J mice infected with four strains of *Echinococcus multilocularis*. (**A**) H&E staining (×100 and ×200). (**B**) Number of foci lesions in the liver samples of the mice (*n* = 6–12 mice per group). (**C**) Liver fibrosis determined by Masson’s staining (×100 and× 200). (**D**) Fibrosis analysis quantified using cellSens Dimension software. (**E**) IHC staining of α-SMA in the mouse liver (×100 and ×200). (**F**) Stained α-SMA quantified by cellSens Dimension software. EM-AK, Alaska strain; EM-JP, Japan strain; EM-XJ, Xinjiang strain; EM-NX, Ningxia strain; H&E, hematoxylin and eosin; α-SMA, α-smooth muscle actin; and Pv: parasitic vesicle. Dashed line marks border of granuloma around parasitic lesion. Blue arrow indicates inflammatory cell zone; red arrow indicates fibrosis areas; and purple arrow indicates α-SMA areas.

Four months after infection, all mice were successfully infected, and most (67–75%) of the mice survived, with few dying before anesthesia was administered. The pathological differences among the four strains are shown in Fig. 2; Table 1 and Fig. S1. The pathological feature of the AE lesions in the liver depended on the size of the lesions. We classified the size of AE lesions into two types: pre-vesicles (PV) (normally, <2 mm in size) and alveolar vesicles (AVs) of metacestodes (size: ≥2 mm). EM-AK had both AV and PV types of lesions, with half having PV (6/12) and the other half having AV + PV (6/12). In contrast, EM-NX had only two out of 10 mice with AV + PV, with most of the lesions (8/10) being AV. EM-XJ had five out of 11 mice with AV + PV. EM-JP had no mice with AV, with all lesions being PV (6/6). The *χ* test analysis indicated a significant difference between EM-AK and EM-JP with types AV + PV (*P* < 0.05). Compared with EM-NX and EM-XJ, there was no significant difference (*P* > 0.05). In terms of the number of lesions in the liver samples, EM-AK (27.58 ± 9.12) had more foci of infection than EM-JP (13.80 ± 2.48) and EM-NX (18.50 ± 3.97), and the difference was significant (EM-AK vs. EM-JP, *P* < 0.01; EM-AK vs. EM-XJ, *P* < 0.05). However, compared with EM-XJ (21.13 ± 5.97), the difference in the lesion numbers of EM-AK and EM-JP was not significant (*P* > 0.05). As shown in Fig. 2A, EM-AK induced a more severe “granuloma” than other strains, and the lesions contained a large number of infiltrated host cells. EM-JP had much fewer foci of infection compared to other strains (Fig. 2B). The decreasing order of severity was as follows: EM-AK > EM-XJ > EM-NX > EM-JP.

**TABLE 1:** Number of infected C57 mice and rate of infection by different strain PSCs via hepatic portal vein^*a*^

Group	No.	Pre-vesicles		Pre-vesicles + alveolar vesicles		*χ*^2^/value^b^	*P* value
		No.	%	No.	%		
EM-AK	12	6	50.00	6	50.00		
EM-JP	6	6	100	0	0	4.500	<0.05
EM-XJ	11	6	54.55	5	45.45	0.4752	>0.05
EM-NX	10	8	80.00	2	20.00	2.121	>0.05

^*a*^ EM-AK, Alaska strain; EM-JP, Japan strain; EM-XJ, Xinjiang strain; EM-NX, Ningxia strain.

^*b*^ EM-AK is compared with EM-JP, EM-XJ, and EM-NX.

### Characteristics of hepatic fibrosis induced by the four *E. multilocularis* strains

Four months after infection, Masson’s staining showed that EM-AK induced a more severe inflammatory response and liver fibrosis than the other three strains (Fig. 2C). The results indicated that EM-AK (27.41 ± 14.13) had a higher percentage of Masson-stained area (%) than EM-JP (3.26 ± 1.92) and EM-NX (7.99 ± 3.38). These differences were statistically significant when compared with EM-JP (*P* < 0.01) and EM-NX (*P* < 0.01) and not significant when compared with EM-XJ (20.43 ± 6.54) (*P* > 0.05) (Fig. 2D). IHC analysis results indicated that EM-AK induced a significantly higher expression of α-SMA around the lesions (Fig. 2E) than the other three strains. The results showed indicated that EM-AK (19.78 ± 5.91) had a statistically significant higher percentage of α-SMA positive cells (%) than EM-JP (6.10 ± 1.59) (*P* < 0.01) and not significant if compared with EM-XJ (14.38 ± 5.70, *P* > 0.05) and EM-NX (12.90 ± 4.12, *P* > 0.05) (Fig. 2F).

### Mitochondrial genome characterization

To compare the differences among the four strains of *E. multilocularis* at the genomic level, the complete mt genomes were sequenced and compared (Fig. S2). The sequence sizes of EM-AK, EM-JP, EM-XJ, and EM-NX were 13,740, 13,737, 13,737, and 13,738 bp, respectively. The annotated sequences were deposited in National Center for Biotechnology Information GenBank under accession numbers OP628492–OP628495.

### Alignment and phylogenetic analysis

**Fig 3:**
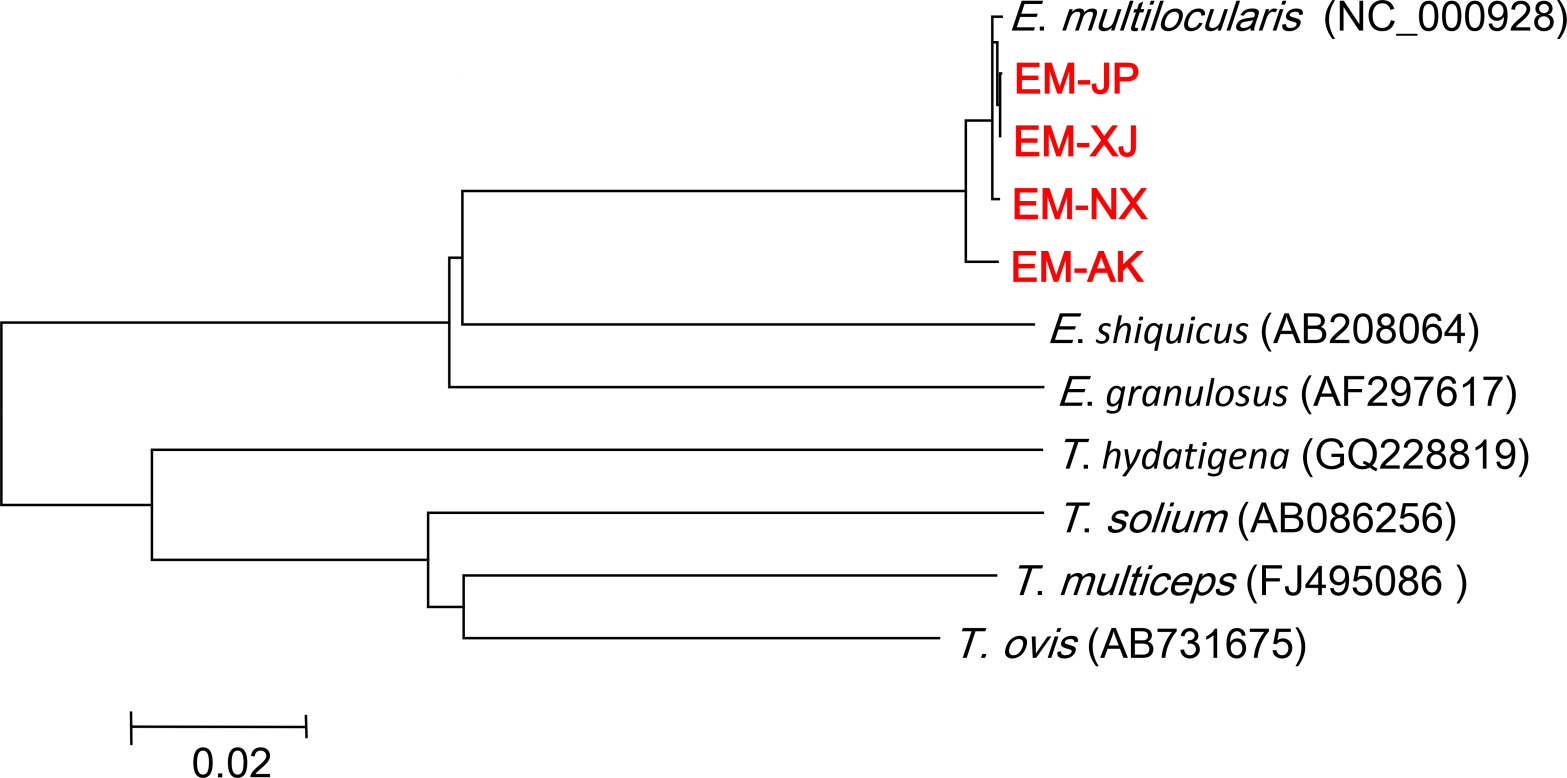
Phylogenetic tree of full mt genomes from four strains of *Echinococcus multilocularis* and two previously published mt genomes of *Echinococcus* and *Taenia* spp. Scale bar represents the estimated number of substitutions per site. The phylogenetic tree was tested by bootstrapping using 1,000 replicates. The red font indicates the four strains reported in this paper. mt, mitochondrial.

**Fig 4:**
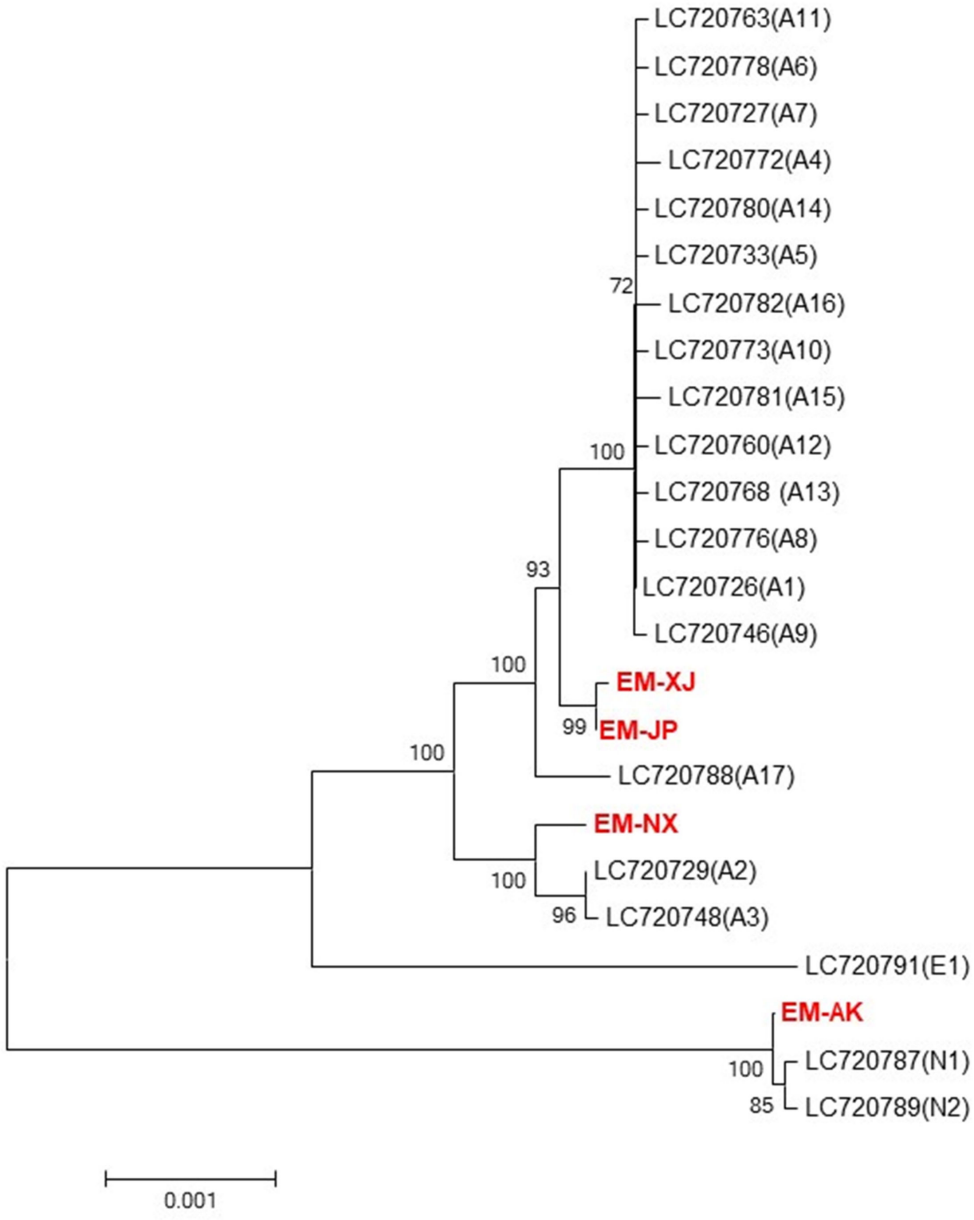
Phylogenetic tree of full mt genomes from four strains of *Echinococcus multilocularis* and 20 previously published *E. multilocularis* mt genomes. The phylogenetic tree was constructed using the neighbor-joining algorithm of the phylogeny program MEGA 10.0. Bootstrap method via 1,000 pseudo replicates was used to assess the reliability of the tree.

Phylogenetic analysis revealed two major clades: the Echinococcus and Taenia clades (Fig. 3). The *E. multilocularis* clade was further subdivided into two clades: one clade comprising EM-AK and the other clade comprising the other three strains, namely, EM-JP, EM-XJ, and EM-NX strain. Based on the topology of our phylogenetic analysis, the Xinjiang strain EM-XJ appeared to be more closely related to the published sequences by Nakao et al. (21). In contrast, EM-AK exhibited the most distant relationship to both EM-XJ and EM-JP, as well as to the published sequences (NC_000928), but showed closer affinity to the published full mt sequences LC720787 1(N1) and LC720789(N2) (22). In another branch, EM-NX was off a small bifurcation and close to Asian 2 (A2) and A3 (Fig. 4). EM-XJ and EM-JP were close to each other (Fig. 3). EM-XJ and EM-JP showed that the full mt sequences were genetically similar to the isolate from Japan, indicating a close evolutionary relationship (Fig. 4). The phylogenetic analysis indicated that EM-AK had a more distant evolutionary relationship from the other three strains with even more genetic distance to *Echinococcus shiquicus*, *Echinococcus granulosus*, *Echinococcus felidis*, and other tapeworms (Table S2). The four sequences were far away from the European sequence 1 (E1), and no similar sequences were found (Fig. 4).

### Molecular clock and phylogenetic tree and network from 22 sequences

**Fig 5:**
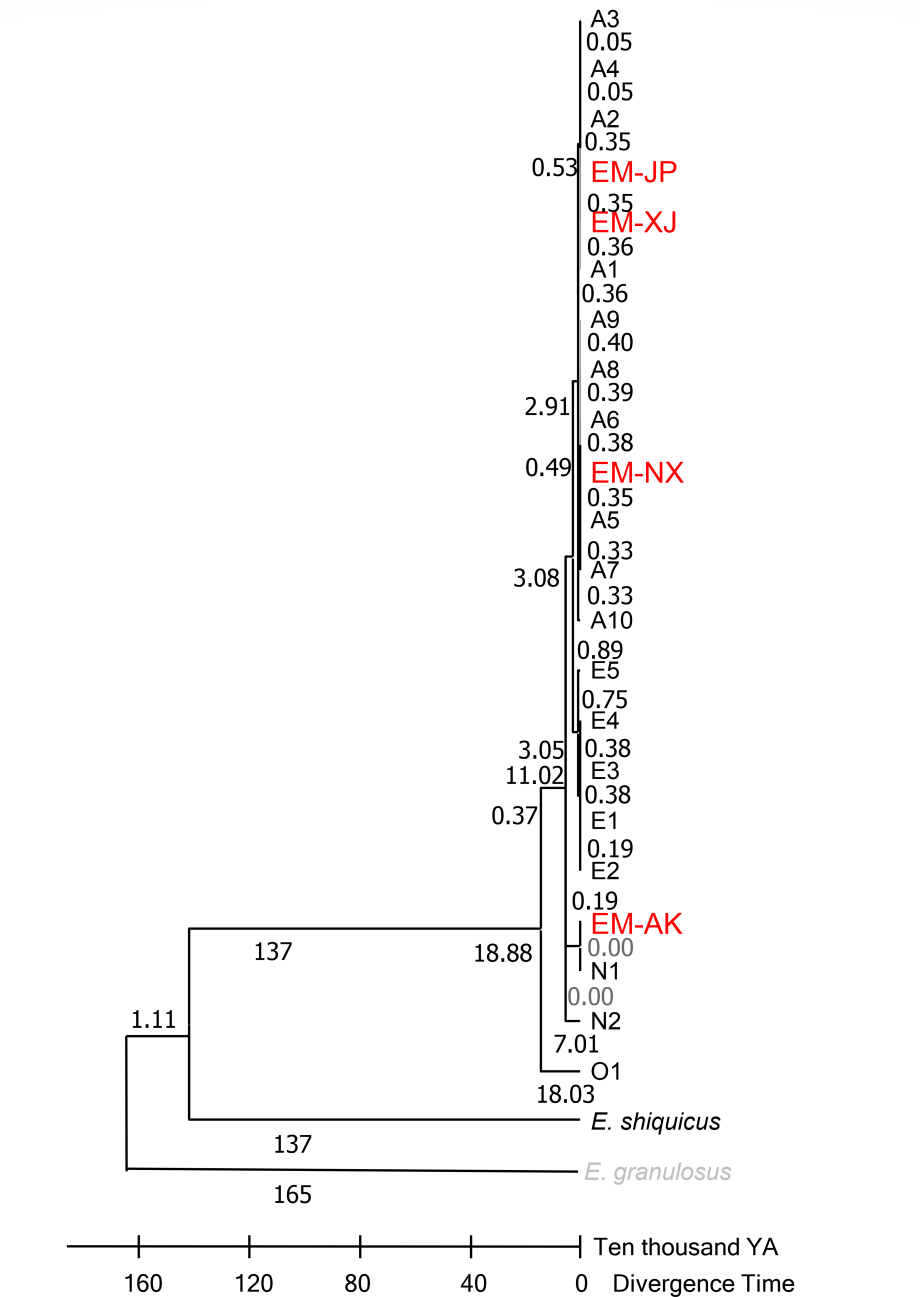
Molecular clock and phylogenetic tree of the concatenated sequences of mitochondrial genes *cob*, *nad2*, and *cox1* from the four strains of *Echinococcus multilocularis* in this study and genes from GenBank. European haplotypes E1–E5, Asian haplotypes A1–A10, and North American haplotypes N1 and N2 are also included, which were originally published in Ref (14). *Echinococcus granulosus* was used as an outgroup.

**Fig 6:**
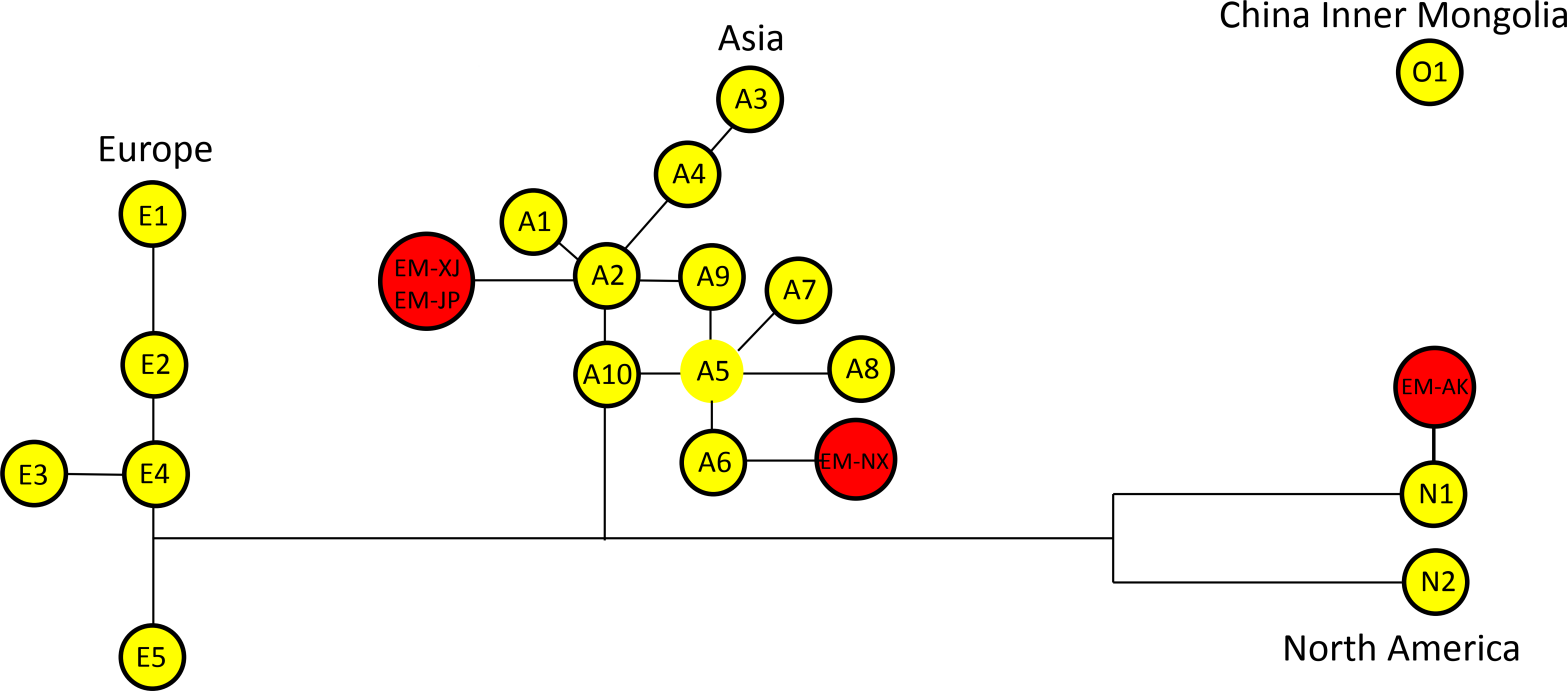
Haplotype network of mitochondrial genes *cob*, *nad2*, and *cox1* of *Echinococcus multilocularis* strains. Concatenated sequences are from GenBank published in Ref. (14), which include European haplotypes E1–E5, Asian haplotypes A1–A10, and North American haplotypes N1 and N2.

Previous studies showed that *E. multilocularis* may be divided into A, E, Inner Mongolian (O), and N geographical clades, which may have occurred 188,800 years ago (14). Our study also evidenced temporal differences between the four strains with bifurcation that occurred 11,020 years ago; among them, the Asian and European strains were very close to each other and separated 29,100 years ago (Fig. 5). In this study, EM-XJ, EM-JP, and EM-NX belong to the Asian/European geographical clade, while EM-AK to the North American clade. EM-XJ was close to EM-JP, which was estimated to have separated 3,500 years ago. EM-JP and EM-NX evolved 29,100 years ago. The molecular clock results are consistent with those of the phylogenetic tree and parsimony network of the time node (Fig. 5 and 6). The evolutionary distance was estimated to have a similar evolutionary result compared with the phylogenetic analysis (Table S3).

## DISCUSSION

### Pathological difference of *E. multilocularis* strains

In this study, we examined four strains of *E. multilocularis* collected from four different geographic regions: one strain from St. Lawrence Island, USA; one from Hokkaido, Japan; and two strains from Ningxia and Xinjiang, China. The original locations of these strains are geographically distant, exhibiting significant geographic isolation. Mt sequences revealed their genetic differences. Importantly, the pathological features observed in the intermediate hosts also differ, especially 4 months after infection. Four months after infection in the peritoneal cavity, EM-AK produced more metacestode weight and induced more pathological responses in mice, followed by EM-XJ, EM-NX, and EM-JP. EM-JP is likely positioned between *E. multilocularis* Alaska and *E. granulosus* in terms of metacestode formation and pathological severity. In addition, the production of PSCs was also different among the strains (Table 2).

**TABLE 2:** Infection rate of the four *Echinococcus multilocularis* strains in intraperitoneally infected KM mice^*a*^

Group	No. of intraperitoneal injection	No. of surviving mice	No. of infected mice	Infection rate (%)	*χ*^2^/value^*b*^	*P* value
EM-AK	10	8	7	87.50		
EM-JP	10	9	5	55.60	2.082	>0.05
EM-XJ	10	8	6	75.00	0.4103	>0.05
EM-NX	10	7	5	71.42	0.6027	>0.05

^*a*^ EM-AK, Alaska strain; EM-JP, Japan strain; EM-XJ, Xinjiang strain; EM-NX, Ningxia strain.

^*b*^ EM-AK is compared with EM-JP, EM-XJ, and EM-NX.

In the intraperitoneal infection KM mice model, EM-AK exhibited granuloma-like larval formations with notably thick outer walls formed by infiltrated host cells. Moreover, EM-AK generated brood capsules that budded off from the germinal layer. Each capsule contains three to 10 PSCs, resembling the characteristics of *E. granulosus* metacetodes.

However, the metacestodes of EM-JP exhibited more transparent cysts with relatively fewer host cells infiltrating around the lesions. In terms of metacestode development, EM-JP formed cyst-like metacestodes that closely resembled the cyst growth of *E. granulosus*. We found that EM-JP produced fewer PSCs than the other three strains. Given the consistent inoculation methodology applied in KM mice, the variance in physiology is likely attributed to genetic disparities among these strains of *E. mulilocularis*.

Interestingly, we found that in the metacestodes of *E. multilocularis*, all isolates can move to other organs, such as the liver, penetrate the diaphragm, and move to the chest cavity, which is different from the cyst growth and development of *E. granulosus* by intraperitoneal innovation (4). This phenomenon may indicate that the larval stage of *E. multilocularis* can be a model for addressing the mechanism of larval migration or parasite metastasis.

Several papers have been published, and the methods of portal vein infection have been reported recognizing that pathological lessons in animal models reflect human pathological responses, so we also adopt this infection method (3.4). The liver infection model was further used to identify the difference among the four strains of *E. multilocularis* in pathology and metacestode growth. Similar to the intraperitoneal infection model, EM-AK induced more severe pathological responses, including more host cell infiltration, causing more tissue damage, thereby inducing more severe necrosis of the liver. The immune response against the larval stages of *E. multilocularis* accounts for, on the one hand, restrained metacestode development and, on the other hand, immunopathological events, eventually leading to liver fibrosis and necrosis among EM-AK, EM-XJ, and EM-NX (4). In particular, EM-AK was the most pathogenic, whereas EM-JP caused fewer occurrences of liver fibrosis and necrosis than the other three strains. This is the first study that comprehensively compared the development and pathological responses between isolates from different continents using the same intermediate host animals.

Clearly, the genotype difference of *E. multilocularis* results in liver pathological difference. It may be impacted on human AE detection and treatment given the pathological difference of AE lesions caused by different strains of *E. mulitlocularis*. We showed previously a three-dimensional image of the liver used for characterization of AE lesions (3, 4). Based on our finding, it is likely that AE caused this.

### Molecular timing of the evolution of *E. multilocularis* strain

In this study, cladistics analysis divided the four *E. multilocularis* strain into Asian and North American geographical clades, reflecting the possibility of isolation during glacial periods. The phylogenetic analysis indicates that the European clade has a sister clade that is close to the Asian clade. The date of bifurcation into the European and Asian clades was estimated to be 30,800 years ago, which is similar to the estimated bifurcation time of 37,000–60,000 years ago by Nakao (14). The genetic distance between EM-AK and EM-JP is relatively large, and these two strains are genetically close to the European and Asian strains, respectively.

The ‘Asian’ geographical clade has been reported in western Russia and St. Lawrence Island, Alaska. Strains of the Asian clade are genetically close to North American N1 sub-strains. Meanwhile, N1 strains have been found in the arctic parts of the Siberia strain (14, 23, 24). This result is related to the phylogenetic analysis, which revealed a geographical clustering pattern (24, 25). Although our sample size is relatively small, it closely aligns with the findings of Bohard et al., who analyzed 72 samples and identified 20 genotypes. Therefore, our four strains can be considered representative of the unique strains in each region, and our sequences correspond to different genotypes, as determined by Bohard et al.’s sequence analysis (Fig. 4).

The bifurcation time is likely associated with the Ice Age and geological, even mainly based on the Ice Age (22). The Asian continent and Japanese islands were even cross-linked with Alaska through the Bering Strait. The ice linkage may have allowed the foxes to be widely distributed, and these infected foxes may have brought *E. multilocularis* to these areas. Then, selection pressures, such as those related to intermediate and definitive hosts, may have accelerated the genome variation, which divided into the European, Asian, and North American geographical clades.

### Phylogenetic analysis showed that EM-AK has far evolutionary distance from other strains of *E. multilocularis*

Mt sequence analysis of 76 geographically distinct *E. multilocularis* strains from Europe, Asia, and North America has clearly delineated three main clades based on geographic distribution, namely, the European, Asian, and North American clades, as described by Nakao et al. (2007) (14). Clearly, EM-AK has a far genetic distance from other strains. These findings were further confirmed by recent report (22), with more strains from different intermediate host species, which showed that A4 has more intermediate host species and are widely distributed (22).

### Intermediate host species are widely distributed and play an important role in genetic variation

Different studies have reported that different species of small mammals are infected with AE in China, including *Arvicola amphibius* (Xinjiang), *Meriones unguiculatus* (Inner Mongolia) (26, 27), *Microtus ilaeus* (Xinjiang, Inner Mongolia, and Sichuan), *Lasiopodomys brandti* (Xinjiang) (28), *Neodon irene* (Eastern Tibetan Plateau), *Microtus musculus* (Xinjiang) (8, 29), *Eospalax fontanierii* (western China) (30), *Ochotona curzoniae* (Sichuan and Qinghai) (29, 31), *Ochotona daurica* (Gansu) (29, 31), and *Spermophilus dauricus* (Ningxia) (32, 33). These intermediate host finding was suggested to cause the genetic variation of *E. multilocularis* (14). *Urocitellus parryii*, *Myodes rutilus*, *Microtus oeconomus*, and *Sorex jacksoni* were also found infected with EM-AK in St. Lawrence Island, North America and gray red-backed vole in Hokkaido, Japan (34, 35). The infection rates by *E. multilocularis* in voles range from 2 to 63% and can reach up to 80% in the spring. Holt et al. found that the *Lemmus trimucronatus* was also infected with metacestode, which is a rare rodent in Alaska, with an infection rate of less than 1% (36)). This introduction resulted in a prevalence of *E. multilocularis* in intermediate hosts, myodes rutilus, as high as approximately 50% on the island (37). However, pathological differences in different hosts have not been reported.

The comparison of mt genetic sequences indicating the difference in the development of the parasite caused the pathology to underline the difference in genetic variation. Sequence analysis is the most important index and the main basis of species classification. We found that previous clusters only relied on *cob nad2* and *cox1* partial or complete sequences (29, 37). Except for the complete mt genome sequences from the two latest reports, which have just published some sequences (17, 22), no other *E. multilocularis* mt genomes have been published in China or in other locations, including Alaska. In this study, the whole mt genomes of *E. multilocularis* from four different geographical regions were compared for the first time, which provides more accurate molecular information, suggesting that EM-AK may be disputed as a different strain, and its taxonomic classification is still debated on.

In the comparative phylogenetic analysis, EM-AK and *E. shiquicus* were relatively close (Fig. 3). Such finding was similarly reported in a previous study (38). *E. shiquicus* was isolated from the Qinghai–Tibetan Plateau, with the adult stage found in Tibetan foxes (*Vulpes ferrilata*) and the larval stage found in plateau pika (*O. curzoniae*) (39). Among the 11 sequences selected for phylogenetic analysis, *E. multilocularis* was closer to *E. shiquicus* than *E. granulosus* (Fig. 3) (39). From the bifurcation point of view, *E. granulosus* is earlier than *E. multilocularis* and *E. shiquicus*, which was similarly reported by Knapp et al. (40). The evolutionary relationship of EM-AK with other Echinococcus species or strains needs further clarification.

## MATERIALS AND METHODS

### Sampling

To determine the pathological difference among the four strains of *E. multilocularis*, KM mice were intraperitoneally inoculated with *E. multilocularis* PSCs collected from differnet geographical areas. The mice were raised in a pathogen-free facility at the First Affiliated Hospital of Xinjiang Medical University (41, 42). These four *E. multilocularis* strains are EM-AK (St. Lawrence Island, Alaska), EM-XJ (Tacheng, Xinjiang), and EM-NX (Xiji, Ningxia) using protocols previously described, and EM-JP collected from Hokkaido, Japan, which was a gift from Prof. Akira Ito from Asahikawa Medical University, Japan many years ago. After being washed five times with phosphate-buffered saline (PBS) (43) at pH = 7.2, the PSCs were digested with 1% (*w*/*v*) pepsin (Sigma-Aldrich, Louis, MO, USA) at pH = 2.0 (adjusted with 2 M of HCl) in Hank’s buffer for 15 min at 37°C. The PSCs were then washed five times with PBS; their viability was determined by staining with 0.1% methylene blue (44); and the dead PSCs were stained in blue. The PSCs with a viability of more than 95% were frozen in liquid nitrogen and stored at −80°C until use.

### Animal infection procedures

Healthy female KM mice (18–22 g) were purchased from the Xinjiang Experimental Animal Center (China). C57BL/6J mice (8–10 weeks old) were purchased from the Beijing Vital River Experimental Animal Technology Co., Ltd. (China). All experimental mice were maintained in pathogen-free animal rooms with a 12 h light/dark cycle and provided with clean food and water. To identify the pathological difference among the four strains of *E. multilocularis* in the liver, we established a liver infection model by injecting PSCs into the liver through the hepatic portal vein of C57BL/6J mice to compare the pathological differences in the liver induced by these strains (44).

To characterize the pathological difference among *E. multilocularis* strains, PSCs were obtained from intraperitoneal lesions maintained in KM mice (*n* = 7–9 mice per group). After being digested with pepsin (solution pH = 2.0) at 37°C for 20 min and washed five times with PBS, pH = 7.2, 5,000 PSCs of each strain were transferred into the peritoneal cavity of KM mice (41, 42). C57BL/6J mice were used for hepatic infection with 2,000 PSCs via the hepatic portal vein (*n* = 6–12 mice per group) (45). Control mice were injected with saline. After 4 months, the mice were sacrificed for pathological tests.

### Pathological staining

For hematoxylin and eosin (H&E) staining and Masson’s staining, the liver samples were fixed with 4% paraformaldehyde. The paraffin-embedded tissues of parasitic lesions and liver samples were sectioned every 4 µm and stained with H&E. The characteristic features of *E. multilocularis* metacestodes and lesions, including the presence of PSCs, the laminated and germinal layers, and host cell infiltrative tissue, were observed under a light microscope (Nikon, Tokyo, Japan). Masson’s staining was processed as in our previous study (45).

### Immunoassay

For immunohistochemical (IHC) analysis, paraffin-embedded liver tissue samples were sectioned as mentioned previously and probed using anti-α-smooth muscle actin (anti-α-SMA) antibody (Abcam, UK) diluted at 1:1,000. The sections were examined microscopically for specific staining, and photographs were taken using a digital image-capture system (Olympus, Tokyo, Japan). The intensity of positive staining was quantified histologically using computer-assisted morphometric analysis (cellSens Dimension software) (Olympus) (46).

### DNA extraction and sequence assembly

Genomic DNA was extracted with the E.Z.N.A. Tissue DNA Kit (Omega Bio-Tek) according to the instructions of the manufacturer. In brief, after digested with pepsin and 10 washes with PBS, about 300 µL precipitated PSCs were soaked in the genomic DNA extraction buffer and homogenized using a microtube mortar and pestle (Sigma, St. Louis, USA). After centrifugation at 12,000×*g* for 3 min, the supernatant was applied to a HiBind DNA spin column to bind DNA. DNA was eluted using 50 µL of water and stored at −80°C for further use.

The complete mt sequences of the four strains of *E. multilocularis* were obtained using high-throughput next-generation sequencing with the paired-end DNA libraries (47) (paired 300 bp reads) constructed and sequenced by CapitalBio Technology using an Illumina MiSeq sequencer (GENterprise GENOMICS, Mainz, Germany). The fragment-sequencing reads were assembled using the mt baiting and iterative mapping algorithm, MITObim 1.8, with the default settings of the program, and the optional quality trimming option trimreads. The full-mt sequences of *E. multilocularis* (GenBank accession number NC_000928) were used as reference sequence for assembly.

### Mt sequence alignment and phylogenetic analysis

The phylogenetic analysis was done using the MEGA 10.0 program. Trees were generated using neighbor-joining (NJ), maximum parsimony (MP), and maximum likelihood (ML) methods based on the Kimura 2-parameter. The robustness of phylogenetic trees was tested by bootstrapping using 1,000 replicates . The alignment of the *E. multilocularis* sequence identity rate was carried out using DNAMAN 7. Parsimony network of mt DNA from 22 concatenated sequences of the genes *cob*, *nad2*, and *cox1* was illustrated by Network 10.2 using statistical parsimony (48).

### Molecular clock

MEGA 10 was used to analyze the evolutionary relationship of 22 concatenated sequences (*cob*, *nad2*, and *cox1*), namely, 10 additional sequences from Asia (A1–A10), five sequences from Europe (E1–E5), two sequences from North America (N1 and N2), and one sequence from China’s Inner Mongolia (O1) . The concatenated sequences of *cob*, *nad2*, and *cox1* were aligned using MUSCLE in MEGA 10. In the tree construction, *E. granulosus* was used as outgroup. The evolution time was predicted using the website http://www.timetree.org/ after maximum and minimum time calibration points were set. The molecular clock was constructed using Kimura’s 2-parameter distances with a gamma shape parameter (a = 0.5). The best-fit nucleotide substitution model was determined for RelTime-ML, a model for calculating the evolution time. In brief, sequence files were prepared for construction of an evolutionary tree with identified nodes using MEGA program. Calibration points were then established using gene divergence times to anchor specific nodes within the tree. Following this, parameters for the RelTime-ML analysis were set. Unlike a strict molecular clock, which assumes constant rates of evolution, RelTime-ML allows for variable rates across different branches of the tree. By applying this method, the divergence times were estimated for each node, and the evolutionary history of the species was deduced.

The robustness of phylogenetic trees was tested by bootstrapping with 1,000 replicates. All parameters were estimated from the data. Gamma distribution with invariant sites (G + I) was used to model evolutionary rate differences among sites. The phylogeny and molecular evolution were simultaneously estimated also using MEGA 10. The GTR model of nucleotide substitution was applied with gamma-distributed rates among sites and a proportion of invariant sites.

### Statistical analysis

Statistical significance was analyzed using GraphPad Prism 7.0 (GraphPad Software, San Diego, CA). Results were expressed as mean ± standard error of the mean. The one-way analysis of variance test comparison was used in PSC productions/number and hepatic fibrosis. The *χ* test was used to compare the differences in intraperitoneally infected/number of infected mice in different groups.

## Data Availability

The mitogenomic sequence is available from GenBank under accession numbers OP628492–OP628495.

## SUPPLEMENTAL MATERIAL

Figure S1 spectrum.01318-24-s0001.tif Size of metacestodes after injection of PSCs via portal vein of four different isolates.

Figure S2 spectrum.01318-24-s0002.tif Nucleotide substitutions of mt of four *E. multilocularis* strains.

Legends spectrum.01318-24-s0003.docx Figure S1 and S2 legends.

Table S1 spectrum.01318-24-s0004.docx Infected organs in mice via intraperitoneal injection of PSCs from different strains of *E. multilocularis*.

Table S2 spectrum.01318-24-s0005.docx The evolutionary distances and nucleotide diversity of full mt genomes of four strains of *E. multilocularis* and two previously published mt genomes of other tapeworms.

Table S3 spectrum.01318-24-s0006.docx The evolutionary distances and nucleotide diversity of the concatenated sequences of the genes *cob*, *nad2*, and *cox1* of four *E. multilocularis* strains and other previously published tapeworms.

ASM does not own the copyrights to Supplemental Material that may be linked to, or accessed through, an article. The authors have granted ASM a non-exclusive, world-wide license to publish the Supplemental Material files. Please contact the corresponding author directly for reuse.
