# Supplementary figures and images for: The genetic variation of mitochondrial sequences and pathological differences of Echinococcus multilocularis strains from different continents
Source: Microbiol Spectr. 2025 Feb 14;13(4):e01318-24. doi: 10.1128/spectrum.01318-24 (PMC11960119; doi:10.1128/spectrum.01318-24)

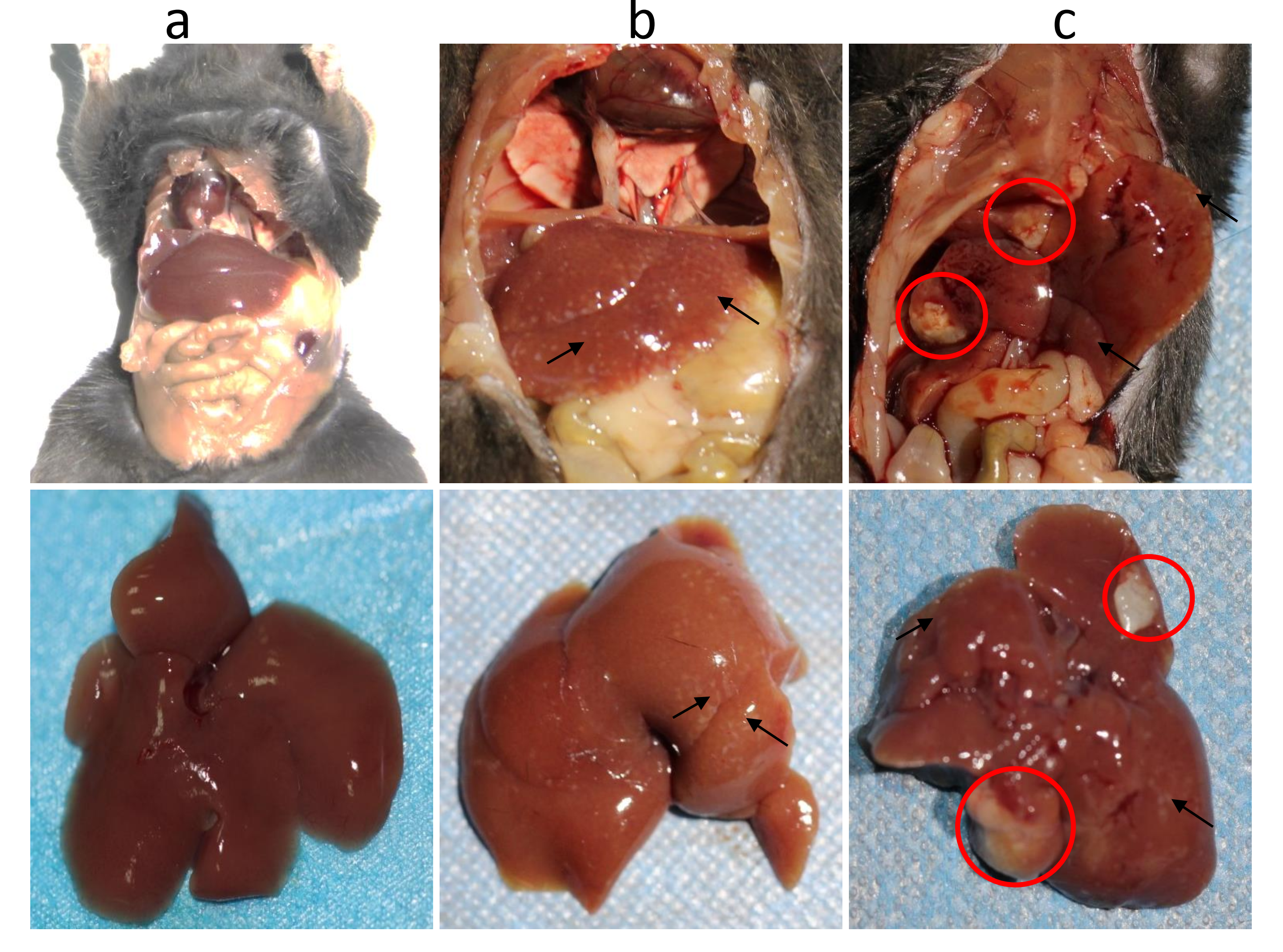

Supplement: Figure S1 — Size of metacestodes after injection of PSCs via portal vein of four different isolates. [file spectrum.01318-24-s0001.tif]

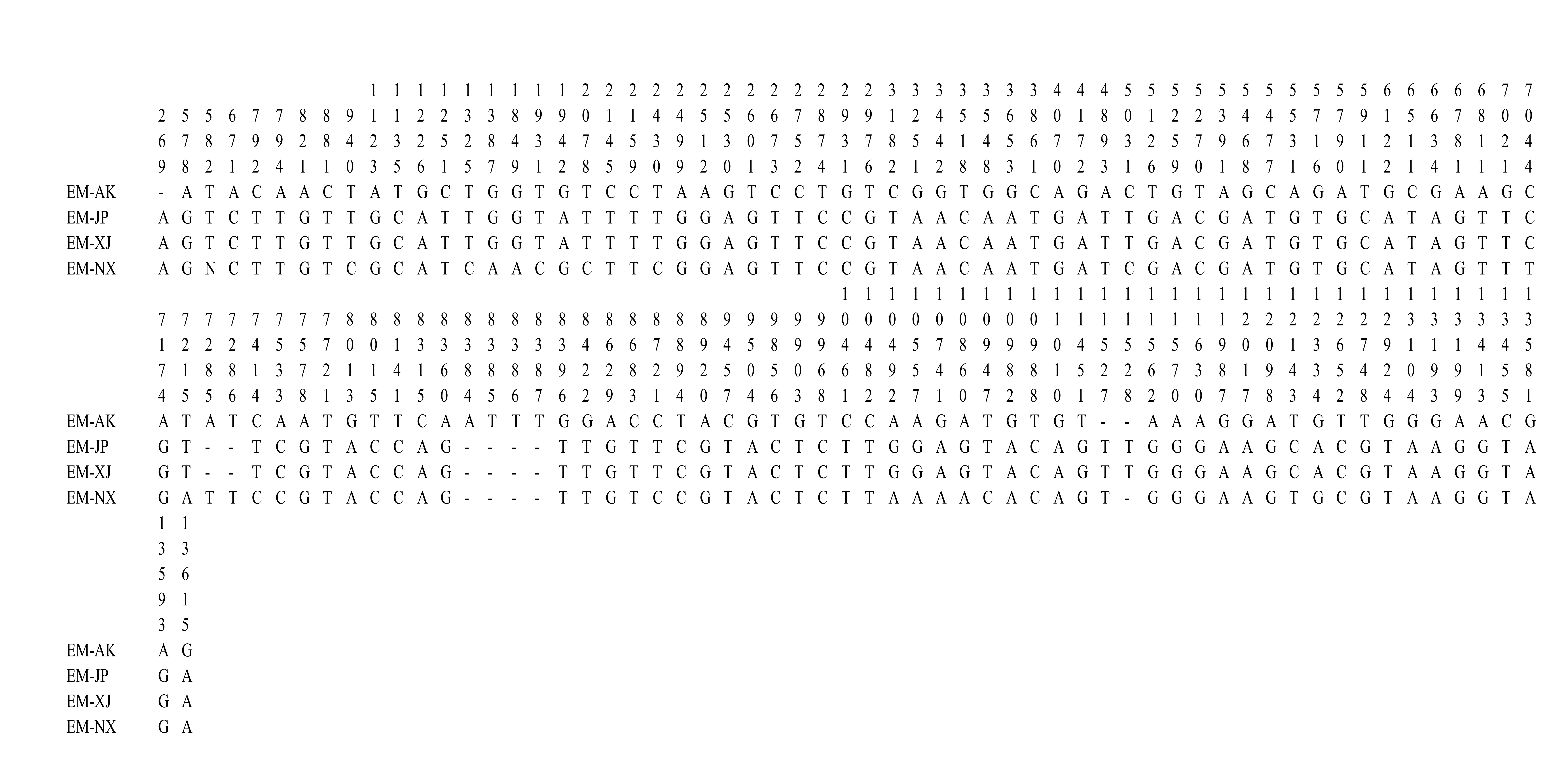

Supplement: Figure S2 — Nucleotide substitutions of mt of four E. multilocularis strains. [file spectrum.01318-24-s0002.tif]
