# Supplementary material for: The genetic variation of mitochondrial sequences and pathological differences of Echinococcus multilocularis strains from different continents
Source: Microbiol Spectr. 2025 Feb 14;13(4):e01318-24. doi: 10.1128/spectrum.01318-24 (PMC11960119; doi:10.1128/spectrum.01318-24)
Supplement: Legends — Figure S1 and S2 legends. [file spectrum.01318-24-s0003.docx]

**Supplementary file 4:** **Figure S1:** Size of metacestodes after injection of PSCs via portal vein of four different isolates. (A) control group, (B) small dots (SD) (normally in size of < 2 mm), (C) SD + alveolar vesicles (AV) of metacestodes (sized ≥ 2 mm.

**Supplementary file 5:** **Figure S2:** Nucleotide substitutions of mt of four *E. multilocularis*. The substitutional sites are totally numbered from the initiation codon of each gene. The numbers are shown in vertical.
