# Supplementary material for: The genetic variation of mitochondrial sequences and pathological differences of Echinococcus multilocularis strains from different continents
Source: Microbiol Spectr. 2025 Feb 14;13(4):e01318-24. doi: 10.1128/spectrum.01318-24 (PMC11960119; doi:10.1128/spectrum.01318-24)
Supplement: Table S1 — Infected organs in mice via intraperitoneal injection of PSCs from different strains of E. multilocularis. [file spectrum.01318-24-s0004.docx]

**Table S1.** Infected organs in mice via intraperitoneal injection of PSCs from different strains of *E. multiolocularis*

| Organs  Strain | Sub mesenteric  adipose tissues | Liver | Spleen | Stomach | Chest cavity | Kidney | Total |
| --- | --- | --- | --- | --- | --- | --- | --- |
| EM-AK | 7 | 6 | 1 | 1 | 1 | 1 | 7 |
| EM-JP | 5 | 4 | 0 | 0 | 0 | 0 | 5 |
| EM-XJ | 6 | 5 | 1 | 0 | 1 | 0 | 6 |
| EM-NX | 5 | 4 | 0 | 1 | 0 | 0 | 5 |
