# Supplementary material for: The genetic variation of mitochondrial sequences and pathological differences of Echinococcus multilocularis strains from different continents
Source: Microbiol Spectr. 2025 Feb 14;13(4):e01318-24. doi: 10.1128/spectrum.01318-24 (PMC11960119; doi:10.1128/spectrum.01318-24)
Supplement: Table S2 — The evolutionary distances and nucleotide diversity of full mt genomes of four strains of E. multilocularis and two previously published mt genomes of other tapeworms. [file spectrum.01318-24-s0005.docx]

**Table S2.** The evolutionary distances and nucleotide diversity of full mt genomes of four strains of *E. multiloculari* and two previously published mt genomes of other tapeworms

| Cestode | NC_000928 | EM-AK | EM-JP | EM-XJ | EM-NX | *E. granulosus* | *E. shiquicus* | *T. hydatigena* | *T. multiceps* | *T. ovis* | *T. solium* |
| --- | --- | --- | --- | --- | --- | --- | --- | --- | --- | --- | --- |
| NC_000928 |  | 0.001 | 0.000 | 0.000 | 0.000 | 0.004 | 0.003 | 0.005 | 0.005 | 0.005 | 0.005 |
| EM-AK | 0.008 |  | 0.001 | 0.001 | 0.001 | 0.004 | 0.003 | 0.005 | 0.005 | 0.006 | 0.005 |
| EM- JP | 0.001 | 0.008 |  | 0.000 | 0.000 | 0.004 | 0.003 | 0.005 | 0.005 | 0.005 | 0.005 |
| EM- XJ | 0.001 | 0.008 | 0.000 |  | 0.000 | 0.004 | 0.003 | 0.005 | 0.005 | 0.005 | 0.005 |
| EM-NX | 0.002 | 0.007 | 0.002 | 0.002 |  | 0.004 | 0.003 | 0.005 | 0.005 | 0.005 | 0.005 |
| *E. granulosus* | 0.131 | 0.131 | 0.131 | 0.131 | 0.132 |  | 0.003 | 0.004 | 0.005 | 0.005 | 0.006 |
| *E. shiquicus* | 0.127 | 0.127 | 0.127 | 0.127 | 0.127 | 0.135 |  | 0.005 | 0.005 | 0.005 | 0.005 |
| *T. hydatigena* | 0.234 | 0.234 | 0.234 | 0.234 | 0.234 | 0.240 | 0.237 |  | 0.004 | 0.004 | 0.004 |
| *T. multiceps* | 0.229 | 0.228 | 0.229 | 0.229 | 0.229 | 0.234 | 0.233 | 0.195 |  | 0.003 | 0.003 |
| *T. ovis* | 0.221 | 0.221 | 0.221 | 0.221 | 0.221 | 0.228 | 0.228 | 0.195 | 0.116 |  | 0.004 |
| *T. solium* | 0.235 | 0.234 | 0.235 | 0.235 | 0.234 | 0.236 | 0.237 | 0.205 | 0.136 | 0.129 |  |
