# Supplementary material for: The genetic variation of mitochondrial sequences and pathological differences of Echinococcus multilocularis strains from different continents
Source: Microbiol Spectr. 2025 Feb 14;13(4):e01318-24. doi: 10.1128/spectrum.01318-24 (PMC11960119; doi:10.1128/spectrum.01318-24)
Supplement: Table S3 — The evolutionary distances and nucleotide diversity of the concatenated sequences of the genes cob, nad2, and cox1 of four E. multilocularis strains and other previously published tapeworms. [file spectrum.01318-24-s0006.docx]

**Table S3.** The evolutionary distances and nucleotide diversity of the concatenated sequences of the genes *cob*, *nad2* and *cox1* of four strains and previously published other tapeworms

|  | EM-AK | EM-JP | EM-XJ | EM-NX | E1 | E2 | E3 | E4 | E5 | A1 | A2 | A3 | A4 | A5 | A6 | A7 | A8 | A9 | A10 | N1 | N2 | O1 | *E. shiquicus* | *E. granulosus* |
| --- | --- | --- | --- | --- | --- | --- | --- | --- | --- | --- | --- | --- | --- | --- | --- | --- | --- | --- | --- | --- | --- | --- | --- | --- |
| EM-AK |  | 0.002 | 0.002 | 0.002 | 0.002 | 0.002 | 0.002 | 0.002 | 0.002 | 0.002 | 0.002 | 0.002 | 0.002 | 0.002 | 0.002 | 0.002 | 0.002 | 0.002 | 0.002 | 0.000 | 0.002 | 0.003 | 0.041 | 0.042 |
| EM-JP | 0.007 |  | 0.000 | 0.000 | 0.001 | 0.001 | 0.001 | 0.001 | 0.001 | 0.000 | 0.000 | 0.001 | 0.000 | 0.000 | 0.001 | 0.001 | 0.001 | 0.000 | 0.000 | 0.002 | 0.002 | 0.003 | 0.039 | 0.041 |
| EM-XJ | 0.007 | 0.000 |  | 0.000 | 0.001 | 0.001 | 0.001 | 0.001 | 0.001 | 0.000 | 0.000 | 0.001 | 0.000 | 0.000 | 0.001 | 0.001 | 0.001 | 0.000 | 0.000 | 0.002 | 0.002 | 0.003 | 0.039 | 0.041 |
| EM-NX | 0.007 | 0.001 | 0.001 |  | 0.001 | 0.001 | 0.001 | 0.001 | 0.001 | 0.001 | 0.000 | 0.001 | 0.001 | 0.000 | 0.000 | 0.000 | 0.001 | 0.000 | 0.000 | 0.002 | 0.002 | 0.003 | 0.038 | 0.041 |
| E1 | 0.009 | 0.005 | 0.005 | 0.005 |  | 0.000 | 0.001 | 0.000 | 0.001 | 0.001 | 0.001 | 0.001 | 0.001 | 0.001 | 0.001 | 0.001 | 0.001 | 0.001 | 0.001 | 0.002 | 0.002 | 0.003 | 0.039 | 0.042 |
| E2 | 0.009 | 0.004 | 0.004 | 0.005 | 0.001 |  | 0.000 | 0.000 | 0.001 | 0.001 | 0.001 | 0.001 | 0.001 | 0.001 | 0.001 | 0.001 | 0.001 | 0.001 | 0.001 | 0.002 | 0.002 | 0.003 | 0.039 | 0.042 |
| E3 | 0.009 | 0.005 | 0.005 | 0.005 | 0.001 | 0.001 |  | 0.000 | 0.001 | 0.001 | 0.001 | 0.001 | 0.001 | 0.001 | 0.001 | 0.001 | 0.001 | 0.001 | 0.001 | 0.002 | 0.002 | 0.003 | 0.039 | 0.042 |
| E4 | 0.009 | 0.004 | 0.004 | 0.004 | 0.001 | 0.000 | 0.001 |  | 0.000 | 0.001 | 0.001 | 0.001 | 0.001 | 0.001 | 0.001 | 0.001 | 0.001 | 0.001 | 0.001 | 0.002 | 0.002 | 0.003 | 0.039 | 0.042 |
| E5 | 0.009 | 0.004 | 0.004 | 0.005 | 0.002 | 0.001 | 0.001 | 0.001 |  | 0.001 | 0.001 | 0.001 | 0.001 | 0.001 | 0.001 | 0.001 | 0.001 | 0.001 | 0.001 | 0.002 | 0.002 | 0.003 | 0.039 | 0.041 |
| A1 | 0.007 | 0.000 | 0.000 | 0.001 | 0.005 | 0.005 | 0.005 | 0.004 | 0.005 |  | 0.000 | 0.001 | 0.001 | 0.001 | 0.001 | 0.001 | 0.001 | 0.000 | 0.000 | 0.002 | 0.002 | 0.003 | 0.039 | 0.041 |
| A2 | 0.007 | 0.000 | 0.000 | 0.001 | 0.005 | 0.004 | 0.005 | 0.004 | 0.004 | 0.000 |  | 0.001 | 0.000 | 0.000 | 0.001 | 0.001 | 0.001 | 0.000 | 0.000 | 0.002 | 0.002 | 0.003 | 0.039 | 0.041 |
| A3 | 0.008 | 0.001 | 0.001 | 0.002 | 0.006 | 0.005 | 0.006 | 0.005 | 0.005 | 0.001 | 0.001 |  | 0.000 | 0.001 | 0.001 | 0.001 | 0.001 | 0.001 | 0.001 | 0.002 | 0.002 | 0.003 | 0.039 | 0.042 |
| A4 | 0.008 | 0.001 | 0.001 | 0.002 | 0.006 | 0.005 | 0.005 | 0.005 | 0.005 | 0.001 | 0.001 | 0.000 |  | 0.001 | 0.001 | 0.001 | 0.001 | 0.001 | 0.001 | 0.002 | 0.002 | 0.003 | 0.039 | 0.042 |
| A5 | 0.007 | 0.001 | 0.001 | 0.000 | 0.005 | 0.005 | 0.005 | 0.004 | 0.005 | 0.001 | 0.001 | 0.002 | 0.002 |  | 0.000 | 0.000 | 0.001 | 0.000 | 0.000 | 0.002 | 0.002 | 0.003 | 0.038 | 0.041 |
| A6 | 0.008 | 0.001 | 0.001 | 0.000 | 0.005 | 0.005 | 0.005 | 0.005 | 0.005 | 0.001 | 0.001 | 0.002 | 0.002 | 0.000 |  | 0.000 | 0.001 | 0.000 | 0.000 | 0.002 | 0.002 | 0.003 | 0.038 | 0.041 |
| A7 | 0.008 | 0.001 | 0.001 | 0.000 | 0.005 | 0.005 | 0.005 | 0.005 | 0.005 | 0.001 | 0.001 | 0.002 | 0.002 | 0.000 | 0.001 |  | 0.001 | 0.000 | 0.000 | 0.002 | 0.002 | 0.003 | 0.039 | 0.041 |
| A8 | 0.008 | 0.002 | 0.002 | 0.001 | 0.006 | 0.005 | 0.006 | 0.005 | 0.005 | 0.002 | 0.002 | 0.003 | 0.003 | 0.001 | 0.001 | 0.001 |  | 0.001 | 0.001 | 0.002 | 0.002 | 0.003 | 0.039 | 0.041 |
| A9 | 0.008 | 0.001 | 0.001 | 0.001 | 0.006 | 0.005 | 0.005 | 0.005 | 0.005 | 0.001 | 0.001 | 0.002 | 0.002 | 0.001 | 0.001 | 0.001 | 0.001 |  | 0.001 | 0.002 | 0.002 | 0.003 | 0.038 | 0.041 |
| A10 | 0.007 | 0.000 | 0.000 | 0.001 | 0.005 | 0.004 | 0.004 | 0.004 | 0.004 | 0.001 | 0.000 | 0.001 | 0.001 | 0.001 | 0.001 | 0.001 | 0.001 | 0.001 |  | 0.002 | 0.002 | 0.003 | 0.039 | 0.041 |
| N1 | 0.000 | 0.007 | 0.007 | 0.007 | 0.009 | 0.009 | 0.009 | 0.009 | 0.009 | 0.007 | 0.007 | 0.008 | 0.008 | 0.007 | 0.008 | 0.008 | 0.008 | 0.008 | 0.007 |  | 0.002 | 0.003 | 0.041 | 0.042 |
| N2 | 0.007 | 0.007 | 0.007 | 0.008 | 0.009 | 0.009 | 0.009 | 0.009 | 0.009 | 0.008 | 0.007 | 0.009 | 0.008 | 0.008 | 0.008 | 0.008 | 0.009 | 0.008 | 0.007 | 0.007 |  | 0.003 | 0.039 | 0.040 |
| O1 | 0.019 | 0.017 | 0.017 | 0.017 | 0.019 | 0.018 | 0.019 | 0.018 | 0.018 | 0.017 | 0.017 | 0.018 | 0.018 | 0.017 | 0.017 | 0.017 | 0.017 | 0.018 | 0.017 | 0.019 | 0.018 |  | 0.039 | 0.042 |
| *E. shiquicus* | 0.152 | 0.147 | 0.147 | 0.147 | 0.150 | 0.149 | 0.150 | 0.149 | 0.149 | 0.148 | 0.147 | 0.148 | 0.148 | 0.147 | 0.147 | 0.148 | 0.148 | 0.148 | 0.147 | 0.152 | 0.147 | 0.150 |  | 0.040 |
| *E. granulosus* | 0.158 | 0.156 | 0.156 | 0.156 | 0.159 | 0.158 | 0.159 | 0.158 | 0.158 | 0.156 | 0.156 | 0.157 | 0.157 | 0.156 | 0.156 | 0.156 | 0.155 | 0.156 | 0.156 | 0.158 | 0.154 | 0.158 | 0.154 |  |
